# A Comprehensive Review on Trends and Patterns of Non-communicable Disease Risk Factors in India

**DOI:** 10.7759/cureus.57027

**Published:** 2024-03-27

**Authors:** Mayank Sharma, Abhay Gaidhane, Sonali G Choudhari

**Affiliations:** 1 Community Medicine, Jawaharlal Nehru Medical College, Datta Meghe Institute of Higher Education and Research, Wardha, IND

**Keywords:** alcohol use, chronic condition, air pollution, unhealthy diet, tobacco use

## Abstract

This review explores the trends and patterns of non-communicable disease (NCD) risk factors in India, with a focus on tobacco use, unhealthy diet, physical inactivity, and air pollution. Drawing upon existing literature and data, the review highlights the substantial burden imposed by NCDs and their associated risk factors on public health and healthcare systems in India. Key findings reveal the widespread prevalence of these risk factors, particularly among certain demographic groups and in urban areas. Socioeconomic disparities also play a significant role in shaping the distribution of NCD risk factors across the population. The review underscores the importance of addressing NCD risk factors through evidence-based interventions and policies tailored to the Indian context. Furthermore, it emphasizes the need for multi-sectoral collaboration among government agencies, healthcare providers, civil society organizations, academia, industry partners, and communities to mitigate the NCD epidemic effectively. By mobilizing collective efforts and resources, India can make significant strides in preventing and controlling NCDs, thereby enhancing population health and well-being.

## Introduction and background

Non-communicable diseases (NCDs) represent a significant health challenge globally, characterized by chronic conditions that are not transmissible from person to person. These diseases encompass a wide range of health conditions, including cardiovascular diseases, cancer, chronic respiratory diseases, and diabetes. Unlike communicable diseases, which often spread through pathogens, NCDs typically develop gradually over time and are strongly influenced by various factors such as lifestyle choices, environmental exposures, and genetic predispositions [1].

India, like many other countries, is undergoing a notable epidemiological transition marked by a notable increase in the prevalence of NCDs. This transition is fueled by several factors, including rapid urbanization, shifts in dietary habits, sedentary lifestyles, and the aging of the population. As a result, there has been a considerable rise in the burden of NCDs across the country. Understanding the risk factors associated with NCDs is paramount for devising effective prevention and control strategies that are tailored to the unique context of India [2].

This review endeavors to delve into the trends and patterns of NCD risk factors, specifically within the Indian context. By amalgamating existing literature and data, the review seeks to shed light on the prevalence, distribution, and determinants of key risk factors such as tobacco use, unhealthy diet, physical inactivity, and air pollution. Moreover, the review will scrutinize the sociodemographic disparities inherent in NCD risk factors, aiming to uncover disparities across different population groups. By doing so, the review aims to offer valuable insights into the implications for public health policy and intervention strategies aimed at tackling the burgeoning challenge of NCDs in India.

## Review

Overview of NCDs in India

Prevalence and Burden of NCDs

In 2017, India witnessed approximately 4.7 million deaths and 226.8 million disability-adjusted life years (DALYs) attributed to NCDs, with a corresponding DALYs to deaths ratio of [3]. NCDs accounted for roughly 5.87 million deaths, representing 60% of all fatalities in the country. Moreover, India accounted for over two-thirds of the total NCD-related deaths in the Southeast Asia Region of the WHO [4]. The primary NCDs prevalent in India encompass cardiovascular diseases, cancer, chronic respiratory diseases, and diabetes, collectively contributing most significantly to NCD-related morbidity and mortality [4]. Alarmingly, the prevalence of NCD risk factors in India, such as tobacco use, unhealthy dietary habits, physical inactivity, harmful alcohol consumption, obesity, elevated blood pressure, heightened blood glucose levels, and increased blood cholesterol levels, has been on the rise. This trend imposes a considerable strain on the healthcare system [4,5]. Notably, per capita consumption of pure alcohol in India is estimated at 5.2 L annually, while more than two-thirds of adolescents aged 11-17 years exhibit sedentary lifestyles [4]. Projections suggest that the upward trajectory in NCD burden will persist in the foreseeable future [6]. Addressing this challenge necessitates a comprehensive, multi-sectoral approach to mitigate NCD risks and implement preventive and control measures [6].

Top NCDs in India Causing Prevalence/Mortality

The primary NCDs in India encompass cardiovascular diseases, chronic respiratory diseases, cancers, and diabetes [4,6]. These diseases collectively represent the majority of NCD-related morbidity and mortality in the country [4,6]. Specifically, cardiovascular diseases, which include conditions such as coronary heart disease, stroke, and hypertension, account for approximately 45% of all NCD deaths in India [7]. Following closely behind are chronic respiratory diseases, contributing to roughly 22% of NCD-related deaths [7]. Cancers and diabetes are also significant contributors, each responsible for around 12% and 3% of NCD-related deaths, respectively [7]. Additionally, other notable NCDs prevalent in India include dementia, hypertension, obesity, and overweight [6].

Economic and Social Impacts of NCDs

The economic and social impacts of NCDs in India are profound. These diseases impose a significant financial burden on individuals and households, leading to a heightened risk of catastrophic out-of-pocket health expenditure, loss of household income, and increased financial insecurity, all of which can exacerbate poverty and inequality [8-10]. Moreover, the economic ramifications of NCDs threaten the achievement of various Sustainable Development Goals (SDGs), notably those related to poverty reduction, inequality reduction, hunger alleviation, access to quality education, and gender equality [6,9]. NCDs also result in productivity losses through premature mortality, early exits from the labor force, absenteeism, and reduced work capacity, posing a substantial obstacle to socioeconomic development [9]. It is estimated that each 10% increase in NCD mortality leads to a 0.5% decrease in annual economic growth [10]. Consequently, the burden of NCDs presents a significant challenge to attaining the SDGs [10]. Moreover, insufficient physical activity is alarmingly high, exceeding 80% among adolescents and 30% among adults in India [6]. Additionally, the burden of NCDs is associated with heightened demand for healthcare services, elevated treatment costs, and mounting pressure for increased public health expenditures, potentially at the expense of broader economic investments [8,9]. Given these substantial economic and social implications, urgent attention and multi-sectoral action are imperative to address the impact of NCDs in India.

Trends and patterns of NCD risk factors

Tobacco Use

Tobacco use stands out as a significant risk factor for NCDs in India, contributing to nearly 1.35 million deaths annually [11]. The country witnesses two primary forms of tobacco consumption: smoking (in the form of bidis, cigarettes, and hookah) and smokeless tobacco (such as khaini, gutkha, betel quid with tobacco, and zarda) [11]. Smokeless tobacco is the most prevalent type, with approximately 267 million adult users, constituting roughly 29% of all adults in India [11]. Concurrently, smoking tobacco affects 10.38% of adults [12]. India grapples with a compressed tobacco epidemic characterized by an upward trend in tobacco use prevalence until 2005-2006, followed by a slight decline from 2005 to 2016 [13]. However, despite this downward trajectory, certain states, particularly those in northern and northeastern regions, continue to report high rates of tobacco use [12,13]. Although men historically exhibit higher rates of tobacco consumption than women, this gender gap is gradually narrowing [12,13]. Several factors influence tobacco use patterns in India, including socioeconomic determinants such as poverty, educational attainment, caste, and geographical region [12,13]. Notably, socially disadvantaged groups, including lower-income individuals, those with lower levels of education, and members of scheduled castes and tribes, demonstrate elevated rates of tobacco use [12]. To combat the burgeoning challenge of tobacco use, the Indian government has instituted various initiatives, including raising taxes on tobacco products, mandating graphic warnings on packaging, and conducting awareness campaigns [14]. However, monitoring and evaluating tobacco use trends is imperative to inform policy decisions and effectively allocate resources [15].

Unhealthy Diet and Nutrition

Unhealthy diet and poor nutrition represent significant risk factors for NCDs like cardiovascular diseases, diabetes, and cancer [16-18]. Globally, an unhealthy diet ranks among the leading contributors to disease burden [16]. Extensive evidence supports the health benefits of a diet rich in whole grains, vegetables, fruits, legumes, and nuts while low in salt, free sugars, and fats, particularly saturated and trans fats [16]. The importance of a healthy diet is emphasized from early life through adequate breastfeeding, which correlates with improved educational outcomes, productivity, and lifelong health [16]. Moreover, a healthy diet aligns with environmental sustainability and is linked to reduced greenhouse gas emissions, less freshwater usage, and lower land mass requirements [16]. However, access to healthy diets remains challenging, especially in low- and middle-income countries and areas afflicted by high rates of food insecurity [16]. The proliferation of highly processed foods, bolstered by aggressive marketing tactics, rapid urbanization, and evolving lifestyles, has led to widespread consumption of unhealthy diets characterized by excessive energy, free sugars, salt, saturated fats, and trans fats [16]. Specific dietary recommendations include increasing the consumption of fruits, vegetables, legumes, nuts, and grains while reducing salt, sugar, and fat intake and opting for unsaturated fats over saturated fats [17]. Addressing dietary habits requires a societal approach, necessitating population-based, multi-sectoral, interdisciplinary, and culturally relevant strategies [17].

Similarly, in the United States, poor dietary patterns were once associated with undernutrition, but today, they predominantly contribute to excess consumption, particularly of calories, saturated fats, trans fats, added sugars, and sodium [19]. Americans typically consume excess calories, saturated fats, trans fats, and added sugars, along with inadequate intake of essential nutrients like vitamin D, calcium, potassium, and fiber [19]. To address these issues, a comprehensive dietary approach emphasizing energy balance, portion control, nutrient density, and inclusion of vegetables, fruits, whole grains, low-fat dairy products, lean protein sources, nuts, seeds, and oils while minimizing solid fats (saturated and trans fats) and added sugars, is recommended [19].

Physical Inactivity

Physical inactivity emerges as a significant risk factor for NCDs in India, with studies reporting varying prevalence rates ranging from 20.3% to 66.8% [20-22]. Notably, the prevalence of physical inactivity tends to be higher among women, literate individuals, and current tobacco users [21]. Various factors influence physical activity behavior, including past exercise habits, perceived self-efficacy, social support, self-confidence, access to facilities, physical environment, gender, and socioeconomic status (SES) [20]. Barriers to regular physical activity participation encompass lower motivation levels, limited free time, fear of falling, financial constraints, transportation issues, pain, and lack of enjoyment [20]. The WHO recommends a cumulative engagement of at least 150 minutes per week of moderate physical activity to enhance physiological functioning, quality of life, and social and work participation [20]. In response to the growing concern, initiatives such as the "Fit India Movement," launched by the government in 2019, aim to promote physical activity nationwide [21,23]. However, despite these efforts, the 2022 India Report Card on physical activity for children and youth revealed that most Indian children fail to meet recommended activity levels, spending most of their day engaged in sedentary behaviors [23].

Harmful Use of Alcohol

The harmful use of alcohol refers to alcohol consumption that results in adverse health, social, and economic outcomes. Globally, alcohol misuse contributes to approximately 7.1% and 2.2% of the total burden of disease for males and females, respectively [24]. Alcohol consumption is associated with a range of health issues, including liver diseases, cancers, cardiovascular diseases, and injuries [24,25]. The risks linked to alcohol use escalate with factors such as the volume of lifetime alcohol consumption, frequency of drinking, and the quantity consumed per occasion [24]. Moreover, the consumption of surrogate and illicitly produced alcohols can introduce additional health risks due to the presence of toxic contaminants [24]. To address the challenge of harmful alcohol use, the WHO advocates for a comprehensive approach involving collaboration across various sectors, including health, finance, transportation, education, agriculture, and urban planning [24]. Among the most cost-effective measures to mitigate harmful alcohol use are raising taxes on alcoholic beverages, implementing restrictions on alcohol advertising, and regulating the physical availability of retail alcohol [24]. Additionally, effective interventions encompass enforcing measures to prevent drunk driving, ensuring access to screening, brief interventions, and treatment programs, and directing efforts toward safeguarding vulnerable populations [24].

Air Pollution

Air pollution is a significant environmental concern in India, with elevated levels of ambient PM2.5 posing a substantial threat to public health and the economy [26,27]. Exposure to PM2.5 is associated with severe health outcomes, including lung cancer, stroke, and heart disease, contributing to an estimated 1.67 million deaths annually in India [26]. The country's primary sources of air pollution include emissions from burning fossil fuels like coal or oil, biomass such as wood and charcoal, crop residues, windblown dust, and industrial activities [26,27]. Notably, over half of PM2.5 emissions in India are generated through a "secondary" process in the upper atmosphere, where various gaseous pollutants from different locations mix and react [26]. Agriculture, industry, power plants, households, and transportation all significantly contribute to the formation of secondary PM2.5 [26]. Given the inherently multi-sectoral and multi-jurisdictional nature of the air pollution challenge in India, a comprehensive "airshed" approach is essential [26]. In response to this pressing issue, India has launched the ambitious National Clean Air Program to reduce particulate matter pollution by 30% by 2024 [2]. Furthermore, enhancing the availability of reliable, timely, and readily accessible data on air pollution represents a crucial area where India can make significant strides [28].

Stress and Mental Health

**Figure 1:**
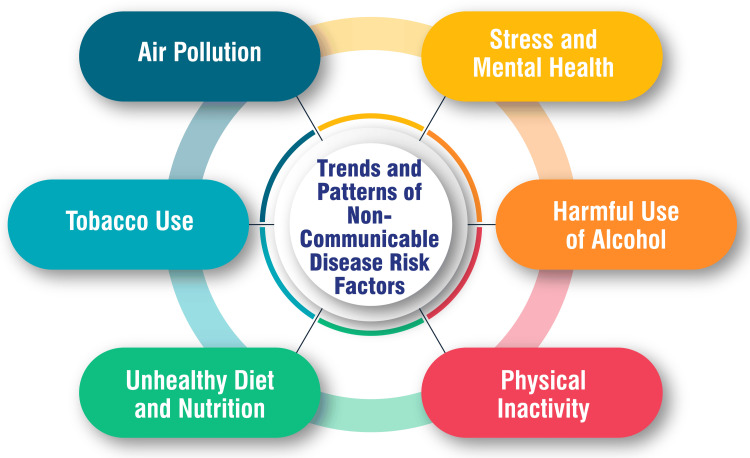
Trends and patterns of non-communicable disease risk factors This figure is self-created by the corresponding author.

Stress and its correlation with mental health represent intricate subjects with multifaceted implications. Prolonged exposure to stress heightens the risk of mental health disorders such as anxiety, depression, substance abuse, sleep disturbances, and physical health complications, including headaches, gastrointestinal issues, and hypertension [29-31]. Stress can manifest across various domains, including cognitive (e.g., difficulty concentrating, memory impairments), emotional (e.g., anxiety, irritability, depression), physical (e.g., headaches, muscle tension), and behavioral (e.g., alterations in eating or sleeping patterns) dimensions [29]. Contributing factors to stress encompass the physical environment, interpersonal relationships, occupational pressures, life circumstances, and significant life transitions [1]. High-risk populations vulnerable to stress-induced mental health issues may include individuals lacking adequate social support, facing multiple stressors, experiencing feelings of hopelessness or helplessness, and encountering adverse life events [29]. Effective stress management entails adopting self-care practices such as maintaining a balanced diet, engaging in regular physical activity, incorporating relaxation techniques, and nurturing a robust support network [29,30]. Moreover, recognizing stress triggers and acquiring coping mechanisms play pivotal roles in averting the progression of stress into chronic states [30]. Trends and patterns of NCD risk factors are shown in Figure 1.

Sociodemographic determinants of NCD risk factors

Age and Gender Trends

Age and gender play pivotal roles in the prevalence and distribution of NCD risk factors, as evidenced by various studies. Research indicates that NCD risk factors tend to escalate with advancing age, with the highest prevalence typically observed among individuals aged 60 years and above, irrespective of gender [32]. However, the interplay between age and NCD risk factors is intricate, with many behavioral risk factors rooted in young adulthood [33]. Regarding gender disparities, studies highlight that males often exhibit higher rates of specific NCD risk factors such as tobacco use, alcohol consumption, and hypertension. At the same time, females tend to have elevated rates of overweight/obesity and hyperlipidemia [32,33]. Additionally, sociodemographic factors, such as educational attainment, level of physical activity, and urbanization status, further influence the prevalence of NCD risk factors [34]. Understanding the age and gender dynamics in NCD risk factors holds paramount importance in formulating effective policies and interventions to mitigate the burden of NCDs. Early diagnosis and treatment of biological risk factors in younger age cohorts are imperative to forestall the manifestation of NCDs later in life. Moreover, behavioral change interventions should target younger age groups to mitigate future NCD development risk [32,33]. By comprehensively addressing age and gender trends in NCD risk factors, stakeholders can devise strategies to promote healthier lifestyles and reduce the incidence of NCDs across diverse demographic groups.

Urban vs. Rural Disparities

As evidenced by numerous studies, disparities in NCD risk factors between rural and urban populations represent a significant public health challenge. Research findings have highlighted variations in NCD risk factors across rural and urban settings. For instance, an analysis of WHO-Strategic Advisory Group of Experts on Immunization (SAGE) data revealed higher prevalence rates of certain NCD risk factors, such as raised waist circumference and diagnosed diabetes, among urban populations. However, exceptions to these trends were noted, such as higher obesity indicators observed in rural regions of Russia and lower rates of active travel reported in urban areas of Ghana and India [35]. Additionally, a recent report from the National Center for Health Statistics highlighted that the age-adjusted death rate in rural areas surpassed that of urban areas by 7% by 2019, with rural regions experiencing a 20% higher death rate compared to urban counterparts [36]. Furthermore, a study conducted in China identified that rural households with NCD patients faced a greater incidence and intensity of catastrophic health expenditure than their urban counterparts [37].

These findings underscore the necessity for tailored strategies to address NCD risk factors in rural and urban settings, accounting for the distinct profiles of each group. Moreover, they emphasize the significance of understanding the sociodemographic determinants influencing NCD risk factors to formulate effective policies and interventions to alleviate the burden of NCDs [35]. By recognizing and addressing rural and urban populations' unique challenges, public health initiatives can strive to reduce NCD-related health disparities and promote equitable access to healthcare resources and services.

SES and Education Levels

**Figure 2:**
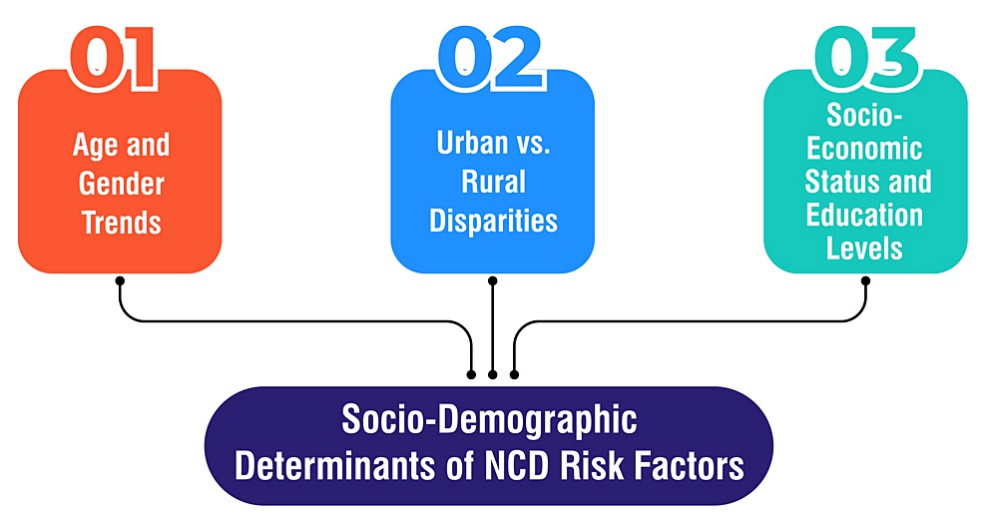
Sociodemographic determinants of NCD risk factors This figure is self-created by the corresponding author.

SES and education levels have emerged as pivotal determinants of NCD risk factors, as elucidated by various studies. For instance, research conducted in Saudi Arabia revealed that lower education levels were linked to a higher prevalence of NCD risk factors, including tobacco use, inadequate fruit and vegetable consumption, low levels of physical activity, overweight/obesity, and hypertension [38]. Similarly, findings from a study in Bangladesh underscored the significant role of household wealth status in determining the overall risk of hypertension and diabetes, with lower SES individuals exhibiting notably lower prevalence rates of these NCD risk factors [39]. Numerous studies across different countries have consistently highlighted the association between SES and NCD risk factors, emphasizing the imperative for targeted interventions to mitigate disparities among various socioeconomic groups [40-42]. These investigations suggest that enhancing education levels and addressing socioeconomic inequalities could substantially alleviate the burden of NCDs. Overall, the evidence underscores the critical role of SES and educational attainment as determinants of NCD risk factors. Addressing these factors is paramount for effectively reducing the burden of NCDs. It is imperative to develop targeted interventions tailored to distinct population groups' socioeconomic and educational backgrounds to effectively mitigate disparities in NCD risk factors. Public health efforts can strive toward fostering health equity and reducing the overall burden of NCDs in communities by prioritizing interventions that account for socioeconomic disparities. Sociodemographic determinants of NCD risk factors are shown in Figure 2.

Policy implications and interventions

Existing Government Policies Addressing NCDs

The Indian government has undertaken significant measures to combat the growing burden of NCDs by developing and implementing strategic frameworks and programs. One such initiative is the National Multisectoral Action Plan (NMAP) for Prevention and Control of Common NCDs (2017-2022), aimed at reducing exposure to key risk factors associated with NCDs. The NMAP outlines ambitious goals to curb tobacco use, promote physical activity, encourage healthy dietary habits, address harmful alcohol consumption, and mitigate air pollution [43]. Additionally, the government has launched the National Programme for Prevention and Control of Cancer, Diabetes, Cardiovascular Diseases, and Stroke (NPCDCS), focusing on enhancing healthcare infrastructure, workforce capacity, health education, early detection, treatment, and referral services [44]. A vital aspect of the NPCDCS is the integration of NCD interventions into the primary healthcare system, emphasizing health promotion and the establishment of NCD clinics at Community Health Centers [44,45]. The National Tobacco Control Programme is crucial in enforcing tobacco control laws nationwide [45]. Furthermore, the government has committed to increasing healthcare expenditure as a percentage of GDP, explicitly focusing on NCD prevention and management [46]. Recognizing the importance of preventive care, there is a concerted effort to shift from a curative approach to a comprehensive strategy to reduce risk factors and foster health-promoting environments [46]. Effective policy formulation and implementation require robust financial support, public-private partnerships, and a multisectoral approach involving various ministries, departments, agencies, and stakeholders [46]. Success hinges on collaborative efforts among domestic and international entities and active involvement from civil society [46]. By leveraging these initiatives and fostering collaboration, the Indian government endeavors to address the multifaceted challenges posed by NCDs and improve its population's overall health and well-being.

Potential Interventions to Reduce NCD Risk Factors

NCDs pose a significant public health challenge in India, necessitating concerted efforts to mitigate associated risk factors. Various interventions have been proposed to address these risk factors and curb the prevalence of NCDs in the country. Firstly, health promotion initiatives are crucial in advocating for healthy lifestyles through health education and behavior change communication [47,48]. Encouraging individuals to adopt healthier habits can substantially reduce their susceptibility to NCDs. Secondly, stringent enforcement of tobacco control laws and implementation of measures, such as increased taxes on tobacco products, is imperative to combat tobacco use, a prominent risk factor for NCDs [6,47]. By discouraging tobacco consumption, the prevalence of NCDs can be significantly reduced. Additionally, promoting physical activity through sports and recreational activities is essential to combat sedentarism, a known risk factor for NCDs [47]. Encouraging people to engage in regular exercise can contribute to improved health outcomes and reduced NCD prevalence. Moreover, advocating for healthy dietary habits, including consumption of diets low in salt, sugar, and saturated/trans-fats, is essential to mitigate the risk of NCDs [6,47]. Dietary interventions play a crucial role in preventing and managing NCDs. Furthermore, efforts to control air pollution, both indoor and ambient, are vital to reducing the risk of NCDs associated with environmental factors [6]. Implementing measures to control emissions and minimize exposure to pollutants can mitigate the burden of NCDs. Similarly, enforcing laws against excessive alcohol consumption and implementing taxation measures are essential components of alcohol control strategies to address alcohol-related NCDs [6,47]. By curbing harmful alcohol use, the prevalence of associated NCDs can be mitigated. Moreover, strengthening infrastructure, human resources, and health systems for early detection, diagnosis, treatment, and referral of NCDs are critical components of preventive efforts [46]. Early intervention can prevent the progression of NCDs and improve health outcomes.

**Figure 3:**
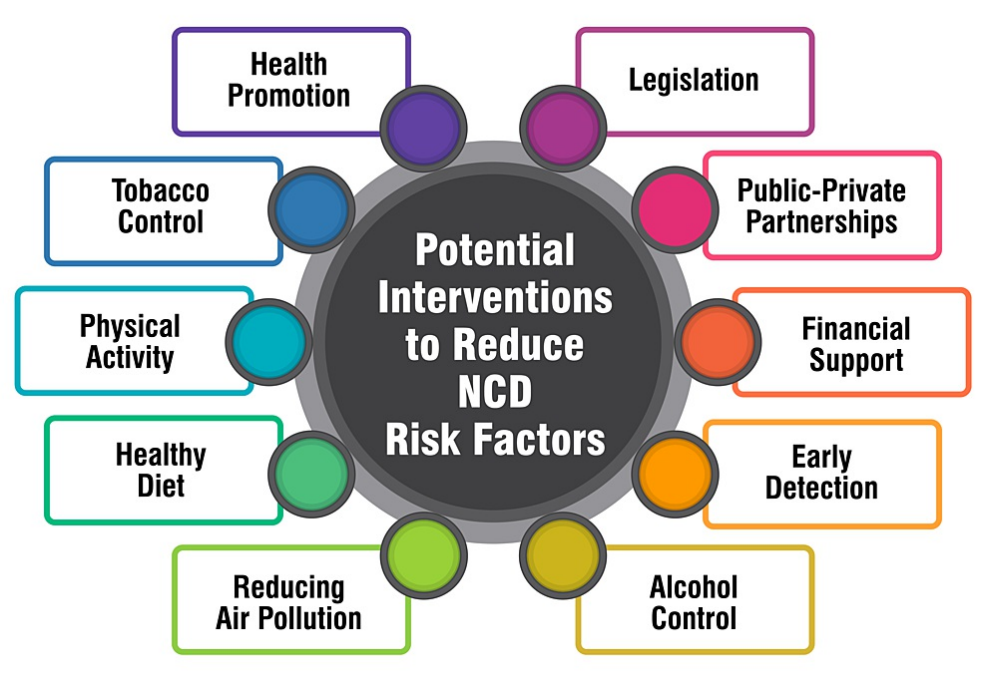
Potential interventions to reduce NCD risk factors This figure is self-created by the corresponding author.

Additionally, prioritizing research to identify effective NCD prevention and control interventions is essential [46]. Developing a national research agenda can guide funding allocation and establish research priorities to address the NCD burden effectively. Financial support is crucial in increasing healthcare spending, mainly targeting NCD prevention and treatment, as a percentage of GDP [47]. Adequate funding is necessary to support comprehensive NCD prevention and control efforts. Furthermore, engaging public-private partnerships involving voluntary organizations and private healthcare sectors in diagnosis, emergency care, and training is vital to enhancing healthcare delivery and capacity building [47]. Lastly, the enforcement of legislation against tobacco usage, excessive alcohol consumption, and unhealthy diets is essential to create a supportive regulatory environment conducive to NCD prevention and control [47]. Stringent enforcement mechanisms can deter risky behaviors and promote healthier lifestyles. Potential interventions to reduce NCD risk factors are shown in Figure 3.

Challenges in Implementing Effective Policies

**Figure 4:**
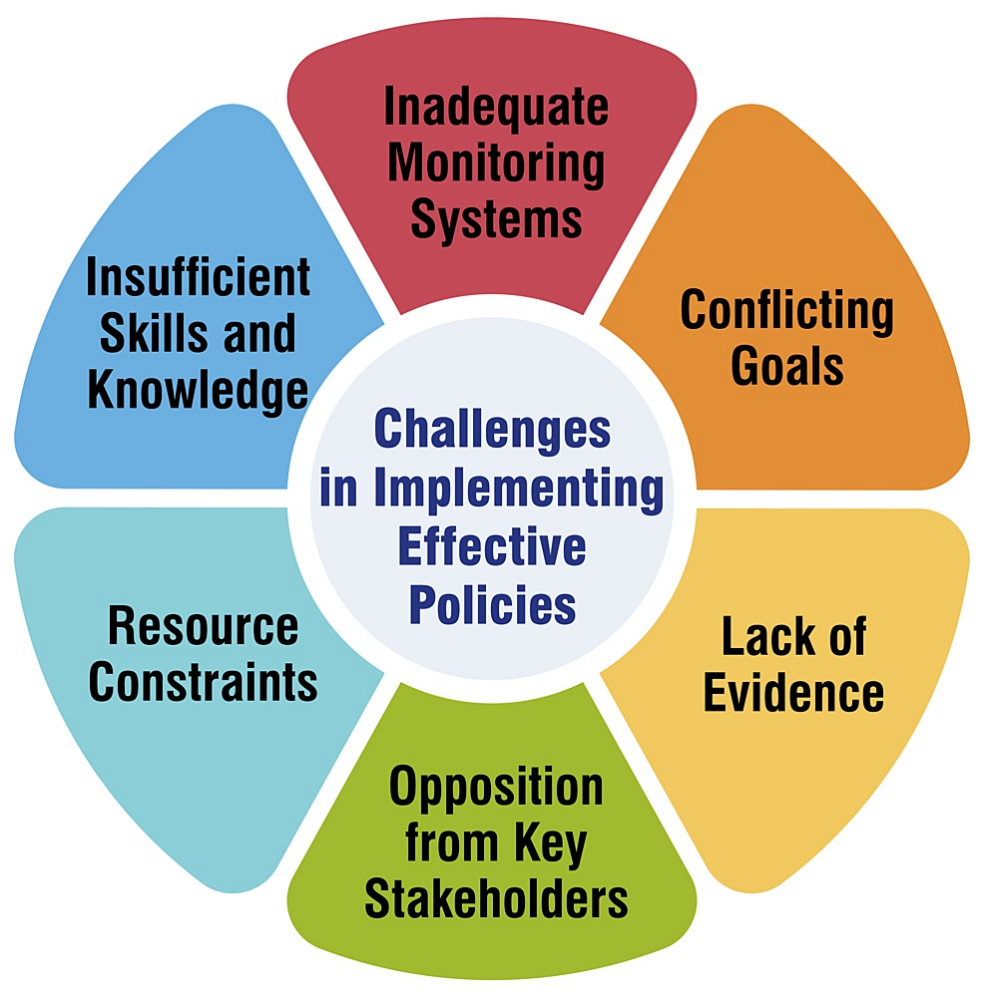
Challenges in implementing effective policies This figure is self-created by the corresponding author.

Implementing effective policies to combat NCDs in India encounters several challenges. Firstly, resource constraints pose significant obstacles, as inadequate financial, material, and human resources can hinder the effective implementation of policies [49,50]. Secondly, opposition from key stakeholders, particularly from industries such as tobacco and alcohol, presents a formidable barrier to policy implementation, often impeding progress [49,51]. Thirdly, more local evidence to support policy initiatives is needed to ensure efforts to address NCDs effectively [49,52]. Moreover, conflicting goals between public health objectives and economic interests can complicate policy implementation, leading to challenges in balancing competing priorities [49,52]. Additionally, the absence of comprehensive monitoring systems to track the implementation of policies poses a significant challenge, as the ability to assess progress and make necessary adjustments needs to be improved [52]. Lastly, inadequate skills and knowledge among implementers may impede effective policy execution, highlighting the need for capacity-building initiatives [49,50]. Addressing these challenges requires concerted efforts to mobilize resources, engage stakeholders collaboratively, generate local evidence, reconcile conflicting interests, establish robust monitoring mechanisms, and provide training and support to enhance implementation capabilities. By overcoming these obstacles, policymakers can enhance the effectiveness of NCD prevention and control strategies, thereby reducing the burden of NCDs on the population. Challenges in implementing effective policies are shown in Figure 4.

Future directions and recommendations

Areas for Further Research

Efforts to address NCDs in India encompass a range of strategies to enhance risk factor assessment, surveillance, intervention efficacy, and equitable access to services. Firstly, there is a need to standardize and expand NCD risk factor assessment nationwide. This involves developing and implementing nationwide protocols utilizing standard WHO NCD risk factor questions in ongoing surveys while broadening the scope of existing surveys to encompass additional risk factors [4]. Secondly, the establishment of an efficient NCD surveillance system is imperative. The design and pilot testing of a cost-effective surveillance system would enable tracking trends in NCDs, facilitating informed decision-making and targeted interventions [4]. Furthermore, efforts should identify effective interventions to strengthen healthcare systems and enhance access to NCD screening, diagnosis, and treatment. Evaluating the impact of government policies and assessing the efficacy of prevention programs targeting tobacco use, environmental improvements, and healthy lifestyles are crucial steps in this regard [44].

In addition, there is a need to explore new research tools and resources to monitor NCDs at the population level, investigate regional variations in NCD risk factors, and understand the role of sociodemographic factors influencing NCD outcomes [44,53]. Moreover, it is essential to evaluate the impact of climate change on NCD risk factors, particularly concerning air quality and temperature extremes. Promoting equity in NCD management by ensuring equitable access to services and resources and focusing on vulnerable populations is another critical aspect of NCD prevention and control efforts [44,53]. Lastly, fostering collaboration among multiple sectors, including health, finance, transportation, education, agriculture, and planning, is essential for comprehensive NCD control. By working together, stakeholders can address the multifaceted determinants of NCDs and implement effective strategies to mitigate their impact [53].

Strategies for Improving NCD Prevention and Control Efforts

**Figure 5:**
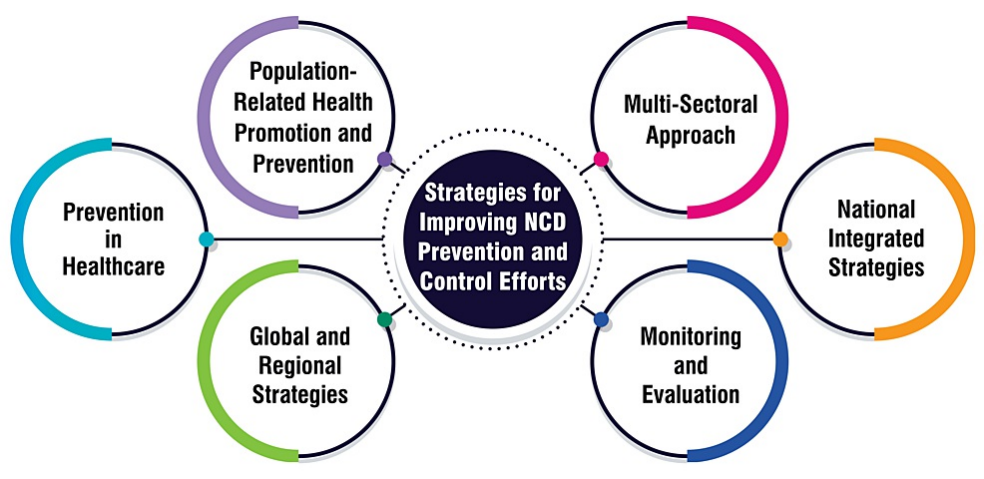
Strategies for improving NCD prevention and control efforts This figure is self-created by the corresponding author.

Efforts to combat NCDs in India encompass a variety of strategies aimed at promoting health and preventing these diseases across different sectors. Firstly, population-related health promotion and prevention initiatives are pivotal. These encompass programs targeting schools, cancer control, trauma prevention, and tobacco control, aiming to promote health and mitigate NCD risk factors at the population level [54]. Secondly, prevention within healthcare settings and integrating NCD prevention and control into healthcare policies across government departments are emphasized. This approach advocates for interventions that address modifiable risk factors shared among NCDs, including tobacco use, physical inactivity, unhealthy diet, and excessive alcohol consumption [55]. A multi-sectoral approach involving collaboration across various health, finance, transport, education, and agriculture sectors is essential. This comprehensive strategy aims to mitigate NCD risks and promote interventions for prevention and control [53]. Furthermore, formulating national integrated strategies for NCD control and prevention is advocated. These strategies should adopt a life-cycle approach, particularly to preventing NCDs in children [56]. Monitoring and evaluation play crucial roles in guiding policy and priorities. Establishing a cost-effective NCD surveillance system and evaluating the implementation of national strategies are essential for tracking progress and trends in NCDs and their risk factors [6,56]. Moreover, India can benefit from global and regional strategies, such as the WHO Global Action Plan for the Prevention and Control of NCDs, and regional plans, such as the "Action Plan for the Prevention and Control of NCDs in the WHO European Region 2016-2025." Adopting and aligning with these strategies can provide valuable frameworks for addressing NCDs effectively [56]. Strategies for improving NCD prevention and control efforts are shown in Figure 5.

Collaboration Opportunities for Public Health Agencies, Non-governmental Organizations, and Communities

Collaboration opportunities among public health agencies, non-governmental organizations (NGOs), and communities are vital for tackling health challenges, including NCDs. NGOs can offer valuable resources such as human and financial assistance, material support, equipment, and communication facilities. At the same time, communities play a crucial role by monitoring functions and safeguarding funds for health centers [57]. Collaboration between the government and NGOs is common in healthcare provision for various communities [58]. However, this collaboration can be intricate due to the involvement of different organizations, each with distinct characteristics and priorities. Individual organizations often focus on their goals and plans, posing challenges to effective collaboration [58]. Therefore, it is imperative to identify the obstacles and issues that hinder collaborative efforts and promote intersectoral collaboration using a systematic approach [58]. The Centers for Disease Control and Prevention (CDC) Foundation spearheads a project to develop inclusive, community-centered recommendations to foster public-private, multi-sector collaborations and system-level responses to ongoing and emerging public health challenges [59]. The final recommendations seek to aid public health agencies in building relationships with community-based organizations (CBOs) to better prepare for and respond to future public health emergencies. Additionally, they aim to promote more equitable solutions in routine practice, effectively advance community health objectives, and share decision-making power with CBOs to address historical disenfranchisement [59]. To ensure that the final recommendations and roadmap for strengthening public-private collaborations are relevant and actionable, they must reflect the diverse experiences of partnerships between public health departments and CBOs across the United States [59]. Thus, engaging representatives from various organizations in various activities, including one-on-one interviews, consensus-building convenings, and insight sessions nationwide, is crucial [59]. Collaboration opportunities among public health agencies, NGOs, and communities are indispensable for addressing health challenges and promoting health equity.

## Conclusions

In conclusion, the review underscores the pressing need to address the pervasive threat of NCD risk factors in India. Key findings highlight the significant burden imposed by factors such as tobacco use, unhealthy diet, physical inactivity, and air pollution, all of which contribute to the escalating incidence of NCDs across the country. Recognizing the detrimental impact of NCDs on individuals, families, and the healthcare system, it becomes imperative for stakeholders to prioritize concerted action. Indeed, tackling NCD risk factors profoundly impacts public health and aligns with broader development goals. Consequently, a collaborative effort among government entities, healthcare providers, civil society organizations, academia, industry partners, and communities is essential. Through evidence-based interventions and policies, from tobacco control measures to initiatives promoting healthy lifestyles and environmental stewardship, stakeholders can effect meaningful change. By embracing this call to action and harnessing collective resources, India can take significant strides toward mitigating the NCD burden, fostering a healthier future for its population.

## References

[1] Al-Hadlaq SM, Balto HA, Hassan WM, Marraiki NA, El-Ansary AK (2022). Biomarkers of non-communicable chronic disease: an update on contemporary methods. PeerJ.

[2] Yadav S, Arokiasamy P (2014). Understanding epidemiological transition in India. Glob Health Action.

[3] Menon GR, Yadav J, John D (2022). Burden of non-communicable diseases and its associated economic costs in India. Soc Sci Humanit Open.

[4] Nethan S, Sinha D, Mehrotra R (2017). Non communicable disease risk factors and their trends in India. Asian Pac J Cancer Prev.

[5] (2024). Non communicable diseases. https://www.who.int/news-room/fact-sheets/detail/noncommunicable-diseases.

[6] (2024). Noncommunicable diseases. https://www.who.int/india/health-topics/noncommunicable-diseases.

[7] Sreeniwas Kumar A, Sinha N (2020). Cardiovascular disease in India: a 360 degree overview. Med J Armed Forces India.

[8] Thakur J, Prinja S, Garg CC, Mendis S, Menabde N (2011). Social and economic implications of noncommunicable diseases in India. Indian J Community Med.

[9] (2024). Economics of NCDs. https://www.paho.org/en/topics/economics-ncds.

[10] Behera S, Pradhan J (2021). Uneven economic burden of non-communicable diseases among Indian households: a comparative analysis. PLoS One.

[11] (2024). Tobacco. https://www.who.int/india/health-topics/tobacco.

[12] Rai B, Bramhankar M (2021). Tobacco use among Indian states: key findings from the latest demographic health survey 2019-2020. Tob Prev Cessat.

[13] Shaikh R, Janssen F, Vogt T (2022). The progression of the tobacco epidemic in India on the national and regional level, 1998-2016. BMC Public Health.

[14] Mishra GA, Pimple SA, Shastri SS (2012). An overview of the tobacco problem in India. Indian J Med Paediatr Oncol.

[15] Rani M, Bonu S, Jha P, Nguyen SN, Jamjoum L (2003). Tobacco use in India: prevalence and predictors of smoking and chewing in a national cross sectional household survey. Tob Control.

[16] (2024). Healthy diet. https://www.who.int/health-topics/healthy-diet.

[17] (2024). NCDs: Unhealthy diet. http://www.emro.who.int/noncommunicable-diseases/causes/unhealthy-diets.html.

[18] (2024). Why Good Nutrition is Important. https://www.cspinet.org/eating-healthy/why-good-nutrition-important.

[19] Institute of Medicine (US) Committee on Examination of Front-of-Package Nutrition Rating Systems and Symbols (2010). Front-of-Package Nutrition Rating Systems and Symbols: Phase I Report. https://pubmed.ncbi.nlm.nih.gov/24983042/.

[20] Mohanty S, Sahoo J, Epari V, Ganesh GS, Panigrahi SK (2022). Prevalence, patterns, and predictors of physical inactivity in an urban population of India. Cureus.

[21] Sharma D, Goel NK, Kaur R, Khosla N, Shekam M (2022). Prevalence and predictors of physical inactivity among adults - a cross-sectional study. Indian J Community Med.

[22] Podder V, Nagarathna R, Anand A, Patil SS, Singh AK, Nagendra HR (2020). Physical activity patterns in India stratified by zones, age, region, BMI and implications for COVID-19: a nationwide study. Ann Neurosci.

[23] Bhawra J, Khadilkar A, Krishnaveni GV, Kumaran K, Katapally TR (2023). The 2022 India report card on physical activity for children and adolescents. J Exerc Sci Fit.

[24] (2024). Harmful use of alcohol. https://www.who.int/health-topics/alcohol.

[25] (2024). Drinking too much alcohol can harm your health. https://www.cdc.gov/alcohol/fact-sheets/alcohol-use.htm.

[26] (2024). Catalyzing Clean Air in India. https://www.worldbank.org/en/country/india/publication/catalyzing-clean-air-in-india.

[27] (2024). India. https://www.cleanairfund.org/geography/india/.

[28] India. AQLI (2024). India. https://aqli.epic.uchicago.edu/country-spotlight/india/.

[29] (2024). Stress. https://www.camh.ca/en/health-info/mental-illness-and-addiction-index/stress.

[30] Contributor WE (2024). What to Know About Stress and How It Affects Your Mental Health. https://www.webmd.com/balance/stress-management/stress-and-how-it-affects-your-mental-health.

[31] (2024). How stress affects your health. https://www.apa.org/topics/stress/health.

[32] Sapkota BP, Baral KP, Rehfuess EA, Parhofer KG, Berger U (2023). Effects of age on non-communicable disease risk factors among Nepalese adults. PLoS One.

[33] Khademi N, Babanejad M, Asadmobini A, Karim H (2017). The association of age and gender with risk factors of noncommunicable diseases among employees in west of Iran. Int J Prev Med.

[34] Pengpid S, Peltzer K (2023). Trends in bio-behavioural risk factors of non-communicable diseases among adults in Sao Tome and Principe. Front Public Health.

[35] Oyebode O, Pape UJ, Laverty AA, Lee JT, Bhan N, Millett C (2015). Rural, urban and migrant differences in non-communicable disease risk-factors in middle income countries: a cross-sectional study of WHO-SAGE data. PLoS One.

[36] (2024). Rural Health Disparities Overview. https://www.ruralhealthinfo.org/topics/rural-health-disparities.

[37] Fu XZ, Sun QW, Sun CQ, Xu F, He JJ (2021). Urban-rural differences in catastrophic health expenditure among households with chronic non-communicable disease patients: evidence from China family panel studies. BMC Public Health.

[38] Al-Hanawi MK, Keetile M (2021). Socio-economic and demographic correlates of non-communicable disease risk factors among adults in Saudi Arabia. Front Med (Lausanne).

[39] Rahman MA (2022). Socioeconomic inequalities in the risk factors of noncommunicable diseases (hypertension and diabetes) among Bangladeshi population: evidence based on population level data analysis. PLoS One.

[40] Allen L, Williams J, Townsend N, Mikkelsen B, Roberts N, Foster C, Wickramasinghe K (2017). Socioeconomic status and non-communicable disease behavioural risk factors in low-income and lower-middle-income countries: a systematic review. Lancet Glob Health.

[41] Sommer I, Griebler U, Mahlknecht P, Thaler K, Bouskill K, Gartlehner G, Mendis S (2015). Socioeconomic inequalities in non-communicable diseases and their risk factors: an overview of systematic reviews. BMC Public Health.

[42] Yaya S, Uthman OA, Ekholuenetale M, Bishwajit G (2018). Socioeconomic inequalities in the risk factors of noncommunicable diseases among women of reproductive age in Sub-Saharan Africa: a multi-country analysis of survey data. Front Public Health.

[43] (2024). National Programme for Prevention and Control of Cancer, Diabetes, Cardiovascular diseases and Stroke (NPCDCS). https://main.mohfw.gov.in/.

[44] Kataria I, Siddiqui M, Gillespie T, Goodman M, Dhillon PK, Bann C, Squiers L (2020). A research agenda for non-communicable disease prevention and control in India. Health Res Policy Syst.

[45] Sinha R, Pati S (2017). Addressing the escalating burden of chronic diseases in India: need for strengthening primary care. J Family Med Prim Care.

[46] Bachani D (2017). Need for strategic revamping to prevent and control non-communicable diseases in India. Indian J Community Med.

[47] Dyson PA, Anthony D, Fenton B (2015). Successful up-scaled population interventions to reduce risk factors for non-communicable disease in adults: results from the International Community Interventions for Health (CIH) Project in China, India and Mexico. PLoS One.

[48] Srivastava RK, Bachani D (2011). Burden of NCDs, policies and programme for prevention and control of NCDs in India. Indian J Community Med.

[49] Jankhotkaew J, Casswell S, Huckle T, Chaiyasong S, Phonsuk P (2022). Barriers and facilitators to the implementation of effective alcohol control policies: a scoping review. Int J Environ Res Public Health.

[50] (2024). Problems of Policy Implementation. https://www.healthknowledge.org.uk/public-health-textbook/medical-sociology-policy-economics/4c-equality-equity-policy/principle-approaches-policy-formation.

[51] Acharya Y, Karmacharya V, Paudel U, Joshi S, Ghimire R, Adhikari SR (2023). Perceptions of key stakeholders on taxes on tobacco and alcohol products in Nepal. BMJ Glob Health.

[52] (2024). What are the challenges of implementing public policies?. https://typeset.io/questions/what-are-the-challenges-of-implementing-public-policies-4su51g4c07.

[53] Ramamoorthy T, Leburu S, Kulothungan V, Mathur P (2022). Regional estimates of noncommunicable diseases associated risk factors among adults in India: results from National Noncommunicable Disease Monitoring Survey. BMC Public Health.

[54] Budreviciute A, Damiati S, Sabir DK (2020). Management and prevention strategies for non-communicable diseases (NCDs) and their risk factors. Front Public Health.

[55] Singh K, Reddy KS, Prabhakaran D (2011). What are the evidence based public health interventions for prevention and control of NCDs in relation to India?. Indian J Community Med.

[56] Gassner L, Zechmeister-Koss I, Reinsperger I (2022). National strategies for preventing and managing non-communicable diseases in selected countries. Front Public Health.

[57] I A Yagub A (2014). Collaboration between Government and Non-Governmental Organizations (NGOs) in Delivering Curative Health Services in North Darfur State, Sudan- a National Report. Iran J Public Health.

[58] Rajabi M, Ebrahimi P, Aryankhesal A (2021). Collaboration between the government and nongovernmental organizations in providing health-care services: a systematic review of challenges. J Educ Health Promot.

[59] (2024). Strengthening Partnerships between Public Health and Community-Based Organizations. https://www.cdcfoundation.org/programs/strengthening-interface-between-public-health-and-community-based-organizations.

